# The role of gender in cognitive outcomes from stroke

**DOI:** 10.1017/S1355617723000036

**Published:** 2023-02-14

**Authors:** Emma Brandt, Sachinkumar Singh, Mark Bowren, Amol Bhagvathi, Daniel Tranel, Aaron D. Boes

**Affiliations:** 1 Department of Psychological and Brain Sciences, University of Iowa, Iowa City, IA 52242, USA; 2 Department of Pediatrics, Carver College of Medicine, University of Iowa, Iowa City, IA 52242, USA; 3 Department of Neurology, Carver College of Medicine, University of Iowa, Iowa City, IA 52242, USA; 4 Department of Psychiatry, Carver College of Medicine, University of Iowa, Iowa City, IA 52242, USA

**Keywords:** gender differences, sex, ischemic stroke, g, IQ, recovery

## Abstract

**Objective:**

Stroke can cause cognitive impairment, which can lead to challenges returning to day-to-day activities. Knowing what factors are associated with cognitive impairment post-stroke can be useful for predicting outcomes and guiding rehabilitation. One such factor is gender: previous studies are inconclusive as to whether gender influences cognitive outcomes post-stroke. Accounting for key variables, we examined whether there are gender differences in cognitive outcomes after stroke.

**Method:**

We analyzed data from neuropsychological assessments of 237 individuals tested in the chronic epoch (≥ 3 months) following ischemic stroke. Using ANCOVA and linear mixed modeling, we examined gender as a predictor of cognition as measured by general cognitive ability (g), Full-Scale IQ, and 18 cognitive tests, controlling for age at stroke onset, education, premorbid intelligence, and lesion volume.

**Results:**

There were no significant gender differences in overall cognitive outcomes as measured by g (*p* = .887) or Full-Scale IQ (*p* = .801). There were some significant gender differences on specific cognitive tests, with women outperforming men on scores from the Rey Auditory Verbal Learning Test (*ps* < .01) and men outperforming women on the Wechsler Adult Intelligence Scale Arithmetic and Information subtests (*ps* < .01).

**Conclusions:**

Our findings indicate that men and women have similar overall cognitive outcomes after stroke, when demographic and lesion factors are accounted for. Although men and women differed in their performance on some individual cognitive tests, neither gender performed systematically better or worse. However, for learning, working memory, and verbal knowledge/comprehension, gender may be an important predictor of outcome post-stroke.

## Introduction

Stroke affects as many as 12 million people each year worldwide (Feigin et al., 2021). Up to 50% of stroke survivors will be chronically disabled, making stroke a leading cause of disability (Donkor, 2018). Cognitive impairment following stroke is an important outcome measure with many downstream effects. More severe cognitive impairment is associated with worse motor function, decreased ability to perform tasks of daily living, and higher mortality (Zietemann et al., 2018). An important goal in stroke research is to develop more accurate prognostic tools to assess one’s risk for cognitive impairment, which may facilitate earlier, targeted interventions. A better understanding of what demographic variables are associated with functional outcomes will be useful in devising models that optimize outcome predictions. Here, we focus on gender as a predictor of cognitive outcome following stroke.

Some previous work has suggested that women have worse functional outcomes following ischemic stroke compared to men (Silva et al., 2010), while other work has suggested that women have a higher likelihood of favorable functional outcome and a lower likelihood of death (Bonkhoff et al., 2021). However, a review of 22 published studies found no consistent gender differences in cognitive outcomes of stroke (Gall et al., 2018). This has prompted questions about whether gender differences, when they are found, might be explained by other factors, such as age, stroke severity, and pre-stroke risk factors (Reeves et al., 2008). For example, in multivariable adjusted studies, gender was not found to significantly contribute to variance in aphasia outcomes after accounting for age (Wallentin, 2018). Similarly, previous work shows that older women may have more severe strokes than older men, which may lead to worse cognitive outcomes (Dehlendorff et al., 2015).

A few important words about terminology: the terms “sex” and “gender” have often been used interchangeably in previous literature examining post-stroke outcomes. However, the two terms are not synonymous. According to the American Psychological Association, “sex” refers to the “biological aspects of maleness and femaleness,” whereas “gender” refers to the “psychological, behavioral, social, and cultural aspects of being male or female” (VandenBos, 2015). In the current study, the appropriate term is “gender,” and we have used that throughout.

### Aims

Despite a large body of work on gender differences in post-stroke cognitive outcomes, the results to date are not definitive. Here, we performed a secondary analysis on a large, extant dataset with participants who have well-characterized cognitive outcomes assessed in the chronic epoch (≥ 3 months post-stroke). Our goal was to evaluate whether men and women differed in cognitive outcome after stroke when accounting for other factors (that may potentially vary by gender), including age at stroke onset, years of education, crystallized intelligence, and lesion volume.

## Methods

Participants were 237 individuals from the Iowa Neurological Patient Registry of the Division of Behavioral Neurology and Cognitive Neuroscience within the Department of Neurology at the University of Iowa. Inclusion criteria included stable focal brain lesion, ischemic stroke as the etiology for the lesion, cognitive testing and structural imaging performed in the chronic epoch (≥ 3 months post-stroke), age of stroke onset 18 years or greater, and the presence of at least 75% of cognitive test data from a neuropsychological test battery. For patients with aphasia, certain cognitive tests could not be administered due to language deficits, and in these cases, we do not report results on these tests for all patients. For any particular patient, which tests were administered was determined by researchers blind to the current study aims. Exclusion criteria (for the Patient Registry generally) include a neurological or psychiatric disorder that preceded the onset of the lesion or a history of significant alcohol or substance abuse. Participants enrolled in the Iowa Neurological Patient Registry completed a large battery of neuropsychological tests. Results of these tests were entered into the Registry database and were available for the current analyses. All participants gave written informed consent to participate in this research, which was approved by the University of Iowa Institutional Review Board, and the research was completed in accordance with the Helsinki Declaration.

Demographic information is provided in Table 1. Gender data were collected via self-report in patient medical records. As alluded to earlier, because participants self-reported their genders and we did not collect any biological information on sex, the focus of this study is gender rather than sex. It should also be noted that until recently, patients were only provided with binary options for gender (male and female) in the standard demographic self-report portal used in our research program. Thus, in the current study, we have only included binary genders. Lesion volume was calculated in cubic millimeters derived from manual segmentation of the three-dimensional anatomy of the lesion in a common template space as described previously (Bowren et al., 2020). Each participant underwent neuropsychological testing according to standard procedures of the Benton Neuropsychology Laboratory (Tranel, 2019).

To examine general cognitive functioning, we utilized two measures of overall cognition. The first was a latent variable estimate of general cognitive ability, g. The process for deriving g was described in an earlier study (Bowren et al., 2020). Briefly, g is a composite score derived from 16 cognitive test scores using structural equation modeling. Prior to entry into the model, test scores were transformed into z-scores to facilitate comparison. Structural equation modeling was performed on the z-scores to produce five composite scores: crystallized intelligence (Gc), visuospatial ability (Gv), learning efficiency (Gl), processing speed (Gs), and working memory (Gwm). A hierarchical model was then used to estimate g from these composite scores. G is calculated as a z-score, with positive values indicating above-average scores and negative values indicating below-average scores (with 0 being exactly average). The second measure of cognition was Full-Scale IQ, which was estimated from the Wechsler Adult Intelligence Scale (WAIS) in a subsample of participants who completed this testing. The WAIS measures a variety of cognitive functions, including verbal comprehension, perceptual reasoning, working memory, and processing speed. Given the range of abilities measured, the WAIS is a well-rounded measure of overall cognitive functioning, and it is highly reliable and valid (Lichtenberger & Kaufman, 2012). Further, performance on the WAIS has been shown to be lower in patients with stroke compared to neurologically healthy adults (Theiling et al., 2013). The WAIS version that was most contemporaneous with the other cognitive testing was used (WAIS-R: n = 41; WAIS-III: n = 90; WAIS-IV: n = 37). When more than one Full-Scale IQ score was measured, we selected the highest score. For two participants, only Verbal IQ was available, and in those two instances, it was used as a proxy for Full-Scale IQ. To examine specific cognitive functions, we utilized 18 cognitive test scores spanning a variety of cognitive functions (see Table 2).

### Statistical analysis

Demographic variables and cognitive outcome scores were summarized in men and women using medians and interquartile ranges as well as means and standard deviations. Group differences between men and women on demographic and lesion variables were examined using independent samples t-tests (age at onset, time since stroke, education level, lesion volume, crystallized intelligence, language tests), chi square tests (lesion laterality), and correlation (lesion location). A power analysis was conducted using G × Power (version 3.1.9.7) prior to analyses. To detect a small effect size with 95% power, 138 participants were needed.

Gender differences were assessed for 20 variables. These included g, Full-Scale IQ, and 18 specific cognitive test scores. Since g and Full-Scale IQ represent composite and full-scale assessments, these variables were analyzed separately using ANCOVA. Multivariate analyses using linear mixed modeling were applied to the specific cognitive test scores.

The linear mixed model analysis that assessed overall gender differences for the specific cognitive test scores included, as fixed effects, gender, cognitive test type, and the gender × cognitive test interaction. The model also included age at stroke onset, years of education, crystallized intelligence, and lesion volume as covariates that allowed for differing effects of the covariate on each specific cognitive test score. An unstructured covariance was assumed in estimating the variance-covariance parameters of the specific cognitive tests scores for each patient. To account for multiplicity, Bonferroni adjusted 95% confidence intervals for the adjusted mean differences and the false discovery rate (FDR) adjusted p-values were used for the 20 variables that were tested.

Prior to running all analyses, histograms and boxplots of the raw data and their residuals were visualized for normality and outliers. No significant deviations from normality or outliers were detected.

## Results

**Figure 1.**
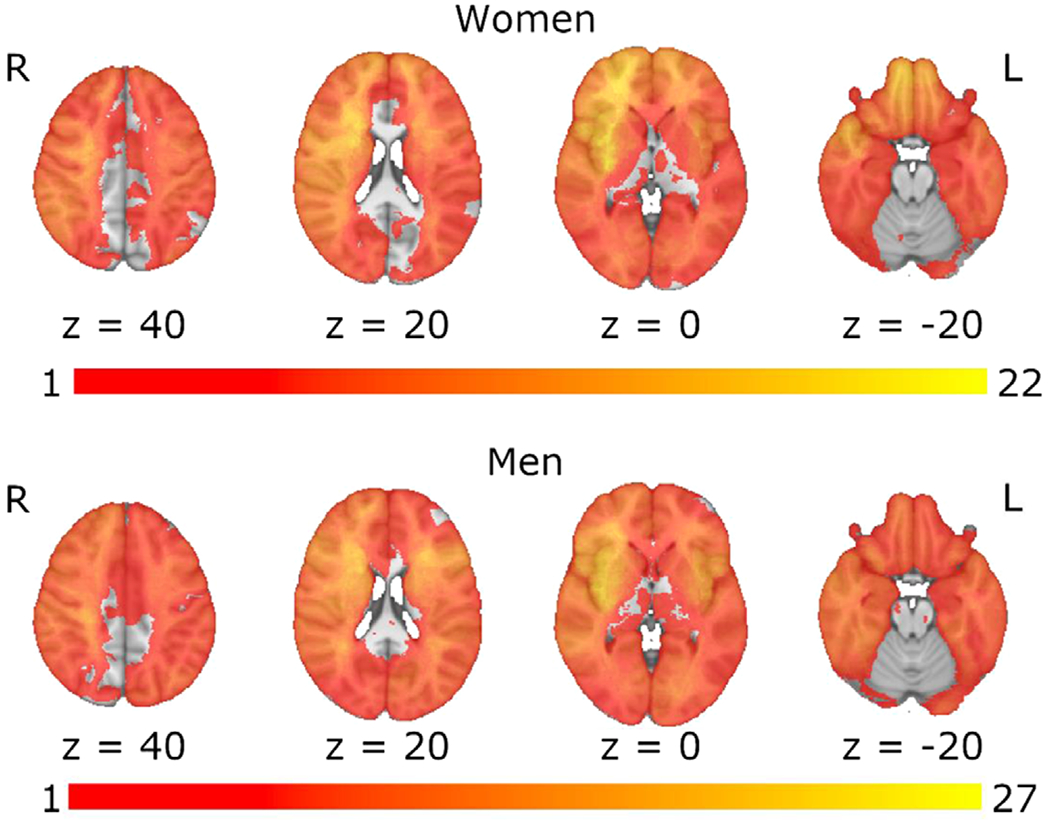
Lesion overlap maps for women and men. Spatial correlation between the lesion overlap maps for women and men: *r* = 0.85 (*p* < .001).

The results from the analysis of demographic variables and primary cognitive outcomes indicated that women were significantly younger at stroke onset (*t*(235) = 2.79, *p* = .006), had significantly fewer years of education (*t*(234.62) = 2.15, *p* = .033), and had significantly lower crystallized intelligence scores (*t*(235) = 2.50, *p* = .013). Men and women showed no significant differences in lesion volume (*t*(235) = 0.98, *p* = .330), time since stroke onset (*t*(235) = −0.76, *p* = .448), or language ability (*t*s < 1.00, *ps* > .200) (Table 1). Additionally, men and women exhibited similar distributions in terms of lesion laterality (chi square = 0.03, *p* = .985), and lesion overlap maps indicated similar lesion coverage in men and women (Figure 1). Table 2 presents medians, interquartile ranges, means, and standard deviations for all cognitive scores.

It should also be noted that (unsurprisingly) g and Full-Scale IQ were highly correlated, with Pearson correlation of *r* = 0.89 (95% CI: 0.85, 0.92). The variable g was also highly correlated with the Arithmetic subtest of the WAIS (*r* = 0.85; 95% CI: 0.80, 0.88) and the Block Design subtest of the WAIS (*r* = 0.71; 95% CI: 0.64, 0.77).

After adjusting for age at stroke onset, years of education, crystallized intelligence, and lesion volume, the mean difference between men and women for g was 0.01, which was not statistically significant (*t*(231) = 0.14, 95% CI: −0.12, 0.14, *p* = .887). The adjusted mean difference between men and women for Full-Scale IQ was 0.33, which was also not statistically significant (*t*(134) = 0.25, 95% CI: −2.27, 2.93, *p* = .801).

The tests of fixed effects from the linear mixed model analyses are shown in Table 3. There was a significant gender × cognitive test interaction (*p* < .001) indicating that the effect of gender on cognitive score differed by specific cognitive test. Likewise, effect of covariates also significantly differed among cognitive tests. Since the effect of gender differed among specific cognitive tests, tests for the difference in mean cognitive score between men and women were assessed for each specific cognitive test. The results from the mixed model analysis are presented in Table 4. Overall, nine specific test scores were significantly different between men and women (FDR adjusted *ps* < .05; Table 5). Women outperformed men on the R-AVLT – trial 5, recall, and delayed recognition hits; WAIS Similarities; and WRAT Word Reading Test. Men outperformed women on the Rey–Osterrieth Complex Figure – recall; and WAIS Arithmetic, Block Design, and Information.

An exploratory analysis was conducted examining patients with left and right lateralized lesions separately. We ran a series of independent samples t-tests comparing men and women on each cognitive measure for patients with left and right lateralized lesions separately. For patients with right lateralized lesions, the R-AVLT, Trail Making Test – Trial B, and the Arithmetic and Information subtests of the WAIS were significantly different for men and women (*ps* < .05). For patients with left lateralized lesions, the R-AVLT and the Block Design and Information subtests of the WAIS were significantly different (*ps* < .05). Thus, men and women differed in their performance on the R-AVLT and the Information subtest of the WAIS regardless of lesion laterality.

## Discussion

We evaluated cognitive outcomes in 237 individuals with ischemic stroke and neuropsychological testing performed in the chronic epoch (≥ 3 months) post-stroke. Our results did not show gender differences in cognitive outcome estimated from g or Full-Scale IQ. These results are consistent with prior suggestions that gender differences in overall cognitive outcomes after stroke may be attributed to other sources of variance, such as differences in age or education (Dong et al, 2020), rather than to gender differences per se. Our results did show gender differences in some demographic and lesion factors, such as age at lesion onset, years of education, and crystallized intelligence, and these could well be potential confounding variables that might contribute to the apparent relationship between gender and overall cognitive outcomes following stroke. It is notable, however, that although statistically significant, the mean difference in years of education between men and women was 0.73 years, which is likely not clinically meaningful. On the other hand, the difference in lesion volume between men and women was not statistically significant but is likely clinically meaningful with a mean difference of 6226.32 cubic millimeters.

In our sample, women outperformed men on the R-AVLT – trial 5, recall, and delayed recognition hits; WAIS Similarities; and WRAT Word Reading Test. Men outperformed women on the Rey–Osterrieth Complex Figure – recall; and WAIS Arithmetic, Block Design, and Information. However, it is noteworthy that only five of those test scores had Bonferroni adjusted confidence intervals that did not include 0 (WAIS Arithmetic, WAIS Information, R-AVLT trial 5, R-AVLT recall, R-AVLT delayed recognition hits). Thus, the most significant differences between men and women were on the R-AVLT (a verbal learning and memory test) and on the WAIS Arithmetic and Information subtests. Although we did observe gender differences on some individual cognitive measures, the fact that men performed better on some measures while women performed better on others argues against a general conclusion that one gender has better (or worse) cognitive outcomes overall following stroke. However, this does suggest that gender may be an important predictor for certain cognitive abilities following stroke, particularly, learning, working memory, and verbal knowledge/comprehension. It is also noteworthy that the gender differences we observed on individual cognitive tests mirrored some findings from studies of gender differences in cognitive functioning in neurologically healthy samples. Specifically, previous work has shown that men tend to perform better on tests of naming, while women tend to perform better on tests of verbal memory and category fluency (Zhang et al., 2017). This suggests that gender differences found in the present study may be reflective of normative gender differences in cognitive functioning, rather than being direct sequelae of stroke.

Additionally, we found some differences between men and women on specific cognitive measures when analyzing patients with left and right lateralized lesions separately. Our results suggest there may be some differences whereby women with right lateralized lesions had lower scores on Trail Making Test – Trial B and the Arithmetic subtest of the WAIS than men with right lateralized lesions. Moreover, women with left lateralized lesions had lower scores on the Block Design subtest of the WAIS than men with left lateralized lesions. A more comprehensive conclusion, however, is that men and women do not have systematically better performances across most measures we studied, at a group level, that would lead to the conclusion that one gender or the other has better cognitive outcomes after stroke.

A strength of the current study is that it included a large cohort of participants who completed an extensive neuropsychological test battery, which enabled us to estimate cognitive ability using two broad measures, g and Full-Scale IQ, and to examine gender differences in several different specific cognitive domains. Additionally, the size of our sample was sufficient to detect relatively small gender differences in post-stroke cognitive ability, so we can have reasonable confidence that our results reflect a true null effect for g and Full-Scale IQ and true differences for measures of learning, working memory, and verbal knowledge/comprehension.

A weakness of this study is the lack of pre-stroke measures of cognitive ability. Cognitive ability prior to stroke has been shown to account for some of the variance in post-stroke outcomes (Dong et al., 2020), and in this sample, this was estimated through education and crystallized intelligence scores. A large population-based cohort that has cognitive testing prior to a stroke would provide a better baseline estimate of cognition and enable researchers to examine relative stroke-related changes in cognition. Furthermore, several of our participants had aphasia, which precluded valid test administration for several cognitive tests. Thus, for some individual tests, such as the WRAT Word Reading Test and the Similarities subtest of the WAIS, our sample sizes were reduced, which may have affected the findings. Further, our findings may not generalize to patients who have aphasia after stroke.

Additionally, here, we focused on self-reported gender and did not include any measures of sex, so we were not able to test the effects of sex on cognitive outcomes of stroke. Some previous work in patients with Alzheimer’s disease has implicated endocrinological differences as a mechanism by which sex differences emerge in cognitive decline (Li & Singh, 2014; Udeh-Momoh & Watermeyer, 2021). Future work could examine whether these endocrinological sex differences could explain differences in cognitive outcomes following stroke. Moreover, we did not include any patients with known non-binary gender (we would note that patients only had a binary self-report option for gender). Future work could examine gender differences in cognitive ability following stroke in a more diverse sample that includes people of other gender identities, such as non-binary and gender fluid.

Another weakness is that our cohort has a high percentage of Caucasian participants (95%), which may limit the generalizability of our findings. Future studies could examine post-stroke gender differences in more diverse samples. Similarly, the mean age of stroke onset for participants in this study was 58.2, which is slightly younger than other samples, which have found mean age of stroke onset to be around 70 (Wang et al., 2013). Thus, although we do not have any reason to believe this affected our findings, the results may not generalize to older stroke samples. Finally, our sample was well-educated (M = 13.4 years of education), so our findings may not generalize to less well-educated samples. This is important because some research has suggested that having attained a higher level of education is associated with greater “cognitive reserve,” which is in turn associated with better cognitive outcomes following stroke (Ojala-Oksala et al., 2012). Despite the restricted variability in terms of education level in our sample, we still did not find gender differences in overall cognitive functioning following stroke after controlling for education. Nonetheless, future work could examine post-stroke gender differences in cognitive ability in samples with a wider range of years of education.

Future work could also examine gender differences in cognitive outcomes from stroke across the chronic epoch. Although we lacked the variability in time since stroke onset to answer this question here, there could be an interaction between gender and time since stroke that would be informative for predicting stroke outcomes. For example, if one gender recovers faster after stroke than the other, the exact timing of post-stroke measurement could affect gender-related findings. This could be important information for clinicians as they try to predict the trajectory of patient outcomes.

## Conclusions

The present study adds to the existing literature on gender differences in cognitive outcomes of stroke. Our findings suggest that there are not significant differences between men and women in overall cognition in the chronic phase of recovery following ischemic stroke. This is in line with some prior research suggesting that other factors besides gender are better predictors of individual differences in stroke outcome (Reeves et al., 2008; Wallentin, 2018). Men and women did differ in their performance on some specific cognitive tests, particularly those involving learning, working memory, and verbal knowledge/comprehension, even after controlling for other factors. Thus, gender may be an important predictor for these particular cognitive functions. This has important implications for how clinicians characterize patient prognoses and approach treatment.

## Figures and Tables

**Table 1.** Demographics

	Women			Men		
	*n*	Median (IQR)	Mean (SD)	*n*	Median (IQR)	Mean (SD)
Age at onset (years)	108	52.6 (42.3–62.4)	52.0 (14.4)	129	58.5 (48.7–65.8)	56.8 (12.2)
Age at scan (years)	108	55.7 (45.6–65.6)	55.3 (14.6)	129	62.1 (52.7–69.1)	60.5 (12.1)
Time since stroke (years)	108	1.4 (0.7–3.9)	3.3 (4.2)	129	1.5 (0.8–5.6)	3.7 (4.4)
Education level (years)	108	12.0 (12.0–14.0)	13.0 (2.3)	129	12 (12–16)	13.8 (2.9)
Lesion volume (mm^3^)	108	15532 (3664–35397)	32541 (54241)	129	24647 (7695–53558)	38767 (43913)
Crystallized intelligence	108	−0.3 (−1.1–0.6)	−0.2 (1.2)	129	0.1 (−0.7–1.3)	0.2 (1.4)
	*n*	Frequency distribution		*n*	Frequency distribution	

Race	105	American Indian – 1 (0.95%)		127	American Indian – 1 (0.8%)	
		African American – 1 (0.95%)			African American – 2 (1.6%)	
		White – 103 (98.10%)			White – 124 (97.6%)	
Handedness	108	Left – 11 (10.19%)		128	Left – 4 (3.2%)	
		Mixed – 5 (4.63%)			Mixed – 6 (4.7%)	
		Right – 92 (85.19%)			Right – 118 (92.9%)	
Lesion laterality	108	Left – 51 (47.22%)		129	Left – 62 (48.1%)	
		Bilateral – 15 (13.89%)			Bilateral – 17 (13.2%)	
		Right – 42 (38.89%)			Right – 50 (38.8%)	
Language function	*n*	Median (IQR)	Mean (SD)	*n*	Median (IQR)	Mean (SD)

Boston Naming Test	49	55.0 (53.0–58.0)	53.6 (6.0)	72	56.0 (53.0–58.0)	52.8 (10.5)
Token Test	49	43.0 (41.0–44.0)	41.7 (3.2)	72	43.0 (40.0–44.0)	40.9 (5.9)
Boston Diagnostic Aphasia Examination – Reading	49	10.0 (9.0–10.0)	9.3 (0.9)	72	10.0 (9.0–10.0)	9.5 (1.4)
Sentence Repetition	49	11.0 (9.0–13.0)	10.8 (2.7)	72	11.5 (9.0–13.5)	10.9 (3.1)

*Note*. IQR = interquartile range, SD = standard deviation.

**Table 2.** General and specific cognitive functioning in patients with ischemic stroke

	Women			Men		
	*n*	Median (IQR)	Mean (SD)	*n*	Median (IQR)	Mean (SD)
Domain-general cognitive ability (g)	108	−0.15 (−0.61–0.40)	−0.15 (0.83)	129	0.15 (−0.63–0.62)	0.06 (0.96)
WAIS Full-Scale IQ	62	95.0 (86.0–105.0)	97.2 (14.3)	78	100.0 (91.0–108.0)	101.2 (15.6)
Benton Facial Recognition Test	97	45.0 (41.0–49.0)	44.8 (5.3)	127	45.0 (40.0–48.0)	43.8 (6.0)
Benton Visual Retention Test – number correct	104	6.0 (5.0–8.0)	6.0 (2.0)	126	6.0 (5.0–7.0)	6.1 (1.9)
Controlled Oral Word Association	105	34.5 (30.0–41.0)	34.7 (11.2)	126	33.5 (23.0–42.0)	32.9 (12.3)
Judgment of Line Orientation	92	24.0 (21.0–28.0)	23.8 (4.9)	122	26.0 (22.0–29.0)	24.8 (5.5)
Rey–Osterrieth Complex Figure – copy	108	30.0 (27.0–33.0)	29.5 (5.1)	128	31.0 (27.8–33.3)	29.5 (5.7)
Rey–Osterrieth Complex Figure – recall	108	16.0 (11.0–20.0)	15.7 (6.2)	129	17.0 (12.0–22.0)	16.8 (7.0)
Rey Auditory Verbal Learning Test (R-AVLT) – trial 5	108	12.0 (10.0–13.0)	11.3 (2.6)	126	10.0 (8.0–11.0)	9.6 (2.9)
Rey Auditory Verbal Learning Test (R-AVLT) – recall	108	9.0 (6.0–11.0)	8.9 (3.5)	126	7.0 (5.0–9.0)	7.0 (3.4)
Rey Auditory Verbal Learning Test (R-AVLT) – delayed recognition hits	108	15.0 (14.0–15.0)	13.9 (2.0)	125	14.0 (12.0–15.0)	12.6 (2.8)
Trail Making Test – Trial A (seconds)	86	36.5 (28.3–49.5)	41.5 (19.1)	111	40.0 (31.0–54.0)	46.8 (22.7)
Trail Making Test – Trial B (seconds)	85	88.0 (64.0–122.0)	102.4 (55.7)	111	99.0 (69.0–169.0)	126.2 (75.4)
WAIS Arithmetic	104	9.0 (7.0–10.0)	8.8 (2.6)	127	10.0 (8.0–13.0)	10.2 (3.2)
WAIS Block Design	106	9.0 (8.0–11.0)	9.1 (2.4)	128	10.0 (8.0–13.0)	10.2 (3.2)
WAIS Digit Span	106	8.0 (7.0–10.0)	8.7 (3.0)	127	8.0 (7.0–10.0)	8.5 (2.9)
WAIS Digit Symbol Coding	107	9.0 (7.0–11.0)	9.0 (2.8)	125	8.0 (6.0–10.0)	8.5 (2.9)
WAIS Information	103	9.0 (8.0–11.0)	9.5 (2.6)	123	11.0 (9.0–13.0)	11.0 (3.1)
WAIS Similarities	97	10.0 (8.0–12.0)	10.1 (2.9)	122	10.0 (9.0–12.0)	10.5 (3.0)
WRAT Word Reading Test	76	97.0 (85.0–104.0)	96.8 (13.4)	98	98.0 (87.3–107.0)	98.5 (14.3)

*Note*. IQR = interquartile range, SD = standard deviation.

**Table 3.** Test of fixed effects from linear mixed model analysis

Type 3 Tests of Fixed Effects				
Effect	df numerator	df denominator	F	*p*-value
Gender	1	212	0.62	.430
Cognitive test	17	212	37.13	< .001
Gender × Cognitive test	17	214	5.16	< .001
Age at lesion onset (years)	1	211	30.32	< .001
Years of education	1	204	3.11	.079
Lesion volume	1	208	5.15	.024
Crystallized intelligence	1	206	0.03	.860
Age at lesion onset × Cognitive test	17	213	9.32	< .001
Years of education × Cognitive test	17	209	2.80	< .001
Lesion volume × Cognitive test	17	213	3.11	< .001
Crystallized intelligence × Cognitive test	17	212	35.25	< .001

**Table 4.** Adjusted mean cognitive score difference between men and women from linear mixed model analysis

Simple differences (men–women) of gender × cognitive test least squares means							
Cognitive test	Adjusted mean difference	Standard error	95% CI		df	t value	*p*-value
Benton Facial Recognition Test	−0.91	0.73	−2.35	0.54	223.6	−1.24	.218
Benton Visual Retention Test	0.26	0.22	−0.18	0.69	227.6	1.17	.243
Controlled Oral Word Association	−2.86	1.38	−5.59	−0.14	226.3	−2.07	.039
Judgment of Line Orientation	0.35	0.67	−0.98	1.67	221.7	0.51	.609
Rey–Osterrieth Complex Figure – copy	0.29	0.64	−0.96	1.54	231.1	0.46	.648
Rey–Osterrieth Complex Figure – recall	2.02	0.77	0.51	3.54	231.0	2.63	.009
Rey Auditory Verbal Learning Test (R-AVLT) – trial 5	−1.54	0.34	−2.21	−0.88	228.0	−4.57	< .001
Rey Auditory Verbal Learning Test (R-AVLT) – recall	−1.70	0.42	−2.53	−0.88	229.7	−4.05	< .001
Rey Auditory Verbal Learning Test (R-AVLT) – delayed recognition hits	−1.05	0.30	−1.65	−0.45	228.1	−3.43	< .001
Trail Making Test – Trial A	1.80	2.72	−3.56	7.16	203.3	0.66	.509
Trail Making Test – Trial B	13.90	7.75	−1.37	29.17	218.8	1.79	.074
WAIS Arithmetic	0.98	0.29	0.40	1.55	227.7	3.34	.001
WAIS Block Design	0.88	0.33	0.22	1.54	228.3	2.63	.009
WAIS Digit Span	−0.76	0.34	−1.43	−0.09	229.9	−2.23	.027
WAIS Digit Symbol Coding	−0.64	0.34	−1.30	0.02	228.3	−1.90	.059
WAIS Information	0.63	0.16	0.31	0.94	230.7	3.90	< .001
WAIS Similarities	−0.47	0.19	−0.85	−0.08	228.3	−2.40	.017
WRAT Word Reading Test	−4.01	1.38	−6.74	−1.28	217.9	−2.89	.004

*Note*. Adjusted for covariates (age at stroke onset, years of education, crystalized intelligence, and lesion volume).

**Table 5.** Bonferroni adjusted 95% confidence interval for the adjusted gender mean score difference and the false discovery rate (FDR) adjusted p-value to account for multiplicity of the 20 variables that were tested

Test	Adjusted mean difference (men–women)	Standard error	Bonferroni adjusted 95% CI	FDR adjusted *p*-value
g	0.01	0.06	−0.19, 0.21	.887
WAIS Full-Scale IQ	0.33	1.31	−3.72, 4.39	.843
Benton Facial Recognition Test	−0.91	0.73	−3.15, 1.34	.311
Benton Visual Retention Test	0.26	0.22	−0.41, 0.93	.324
Controlled Oral Word Association	−2.86	1.38	−7.09, 1.36	.071
Judgment of Line Orientation	0.35	0.67	−1.72, 2.41	.717
Rey–Osterrieth Complex Figure – copy	0.29	0.64	−1.65, 2.23	.720
Rey–Osterrieth Complex Figure – recall	2.02	0.77	−0.32, 4.37	.023*
Rey Auditory Verbal Learning Test (R-AVLT) – trial 5	−1.54	0.34	−2.58, −0.51	< .001**
Rey Auditory Verbal Learning Test (R-AVLT) – recall	−1.70	0.42	−2.99, −0.42	< .001**
Rey Auditory Verbal Learning Test (R-AVLT) – delayed recognition hits	−1.05	0.30	−1.98, −0.12	.004**
Trail Making Test – Trial A	1.80	2.72	−6.52, 10.12	.636
Trail Making Test – Trial B	13.90	7.75	−9.79, 37.60	.114
WAIS Arithmetic	0.98	0.29	0.08, 1.87	.004**
WAIS Block Design	0.88	0.33	−0.14, 1.90	.023*
WAIS Digit Span	−0.76	0.34	−1.80, 0.28	.053
WAIS Digit Symbol Coding	−0.64	0.34	−1.67, 0.39	.098
WAIS Information	0.63	0.16	0.13, 1.12	< .001**
WAIS Similarities	−0.47	0.19	−1.06, 0.13	.038*
WRAT Word Reading Test	−4.01	1.38	−8.24, 0.23	.014*

*Note*. Adjusted for covariates (age at stroke onset, years of education, crystalized intelligence, and lesion volume).

* Indicates *p* < .05.

** Indicates *p* < .01 and 95% CI does not include 0.
